# Insulin Controls Clock Gene Expression in the Liver of Goldfish Probably via Pi3k/Akt Pathway

**DOI:** 10.3390/ijms241511897

**Published:** 2023-07-25

**Authors:** Nuria Saiz, Cristina Velasco, Nuria de Pedro, José Luis Soengas, Esther Isorna

**Affiliations:** 1 Department of Genetics, Physiology and Microbiology, Faculty of Biological Sciences, Complutense University of Madrid, 28040 Madrid, Spain; nursaiz@ucm.es (N.S.); ndepedro@bio.ucm.es (N.d.P.); 2 Centro de Investigación Mariña, Laboratorio de Fisioloxía Animal, Departamento de Bioloxía Funcional e Ciencias da Saúde, Facultade de Bioloxía, Universidade de Vigo, 36310 Vigo, Spain; cvrubial@uvigo.es (C.V.); jsoengas@uvigo.gal (J.L.S.)

**Keywords:** circadian system, food-entrainable oscillator (FEO), *Carassius auratus*, insulin, liver, period genes, phosphatidylinositol 3-kinase (PI3K), fish

## Abstract

The liver circadian clock plays a pivotal role in driving metabolic rhythms, being primarily entrained by the feeding schedule, although the underlying mechanisms remain elusive. This study aimed to investigate the potential role of insulin as an intake signal mediating liver entrainment in fish. To achieve this, the expression of clock genes, which form the molecular basis of endogenous oscillators, was analyzed in goldfish liver explants treated with insulin. The presence of insulin directly increased the abundance of *per1a* and *per2* transcripts in the liver. The dependency of protein translation for such insulin effects was evaluated using cycloheximide, which revealed that intermediate protein translation is seemingly unnecessary for the observed insulin actions. Furthermore, the putative interaction between insulin and glucocorticoid signaling in the liver was examined, with the results suggesting that both hormones exert their effects by independent mechanisms. Finally, to investigate the specific pathways involved in the insulin effects, inhibitors targeting PI3K/AKT and MEK/ERK were employed. Notably, inhibition of PI3K/AKT pathway prevented the induction of *per* genes by insulin, supporting its involvement in this process. Together, these findings suggest a role of insulin in fish as a key element of the multifactorial system that entrains the liver clock to the feeding schedule.

## 1. Introduction

The circadian system integrates periodic environmental cues and anticipates them, maintaining a temporal organization of physiological, behavioral, and metabolic functions [1]. It is composed by a network of endogenous clocks that are based on cell-autonomous transcriptional–translational feedback loops (TTFLs) of clock gene expression with a period of about 24 h [2,3]. The positive limb of the core clock consists of the products of the genes *bmal1* (*arnt1*) and *clock*, which form the BMAL1:CLOCK heterodimer and activate the transcription of genes containing E-box sites in their promoter. These induced genes include *per* and *cry*, which together constitute the negative limb of the core clock, forming PER:CRY complexes. In turn, PER:CRY interacts with BMAL1:CLOCK and inhibits its transcription-enhancing activity. Subsequently, PER and CRY levels drop, and the cycle is restarted [2,3,4]. There are other TTFLs that stabilize the core clock, known as auxiliary loops. One of them includes the nuclear receptors REV-ERBα and RORα, which are also induced by CLOCK:BMAL1 as they contain E-boxes. At the same time, they regulate *bmal1* transcription by repressing or enhancing it, respectively [2,5]. These loops ultimately drive the rhythmic transcription of a great number of clock-controlled genes (CCGs), generating the outputs of the circadian system, i.e., physiological and behavioral rhythms [6]. The circadian system of teleost fish shares most of its architecture with that of mammals, with some differences [4,7,8]. In both taxa, clock gene oscillations occur in virtually all body tissues [9]. However, in mammals there is one main pacemaker in the suprachiasmatic nucleus (SCN) that coordinates the rest, whereas in teleosts the oscillators communicate with one another in a less hierarchical network [9,10]. In addition, teleosts have a higher number of copies of each clock gene, due to additional whole genome duplication events [8,11].

In order to sustain circadian rhythms, endogenous oscillators need to be periodically entrained by cyclic cues known as synchronizers or *Zeitgebers* [12]. Undoubtedly, the main synchronizer of the circadian system is the daily light/dark cycle [12,13], although the fasting/feeding cycle also seems to be important [14,15]. In teleost fish, which have a less-hierarchical circadian system than mammals, light is considered the main *Zeitgeber* [9,10]; it can even directly phase shift the clock, as demonstrated in several zebrafish cell types in culture [4,9]. This suggests that the different tissue clocks are independently entrained and can respond directly to different synchronizing cues [8,10]. Furthermore, in vivo, most oscillators in the fish central nervous system are dependent on the photocycle and are, therefore, classified as light-entrainable oscillators (LEOs) [16,17]. Meanwhile, in peripheral organs, and specifically in the digestive system, clock genes are preferentially entrained by food availability [8,10]. Therefore, such organs could be mainly considered food-entrainable oscillators (FEOs), although they can also respond to other signals [8,10].

In mammals, the liver clock seems to have high physiological relevance, since an estimated 16% of the liver transcriptome and 20% of its proteome is expressed in a circadian manner [6]. The liver is one of the tissues with more oscillating transcripts, including nutrient transporters, receptors, and enzymes [6]. Considering that this organ plays a central role in all metabolic processes in the organism, this indicates that the hepatic core TTFL drives many metabolic cycles [18]. It is, therefore, not surprising that the proper functioning of the liver circadian pacemaker is considered a prerequisite for metabolic health, ensuring an optimal temporal sequence of metabolic processes [18]. The liver oscillator is one of the pacemakers that most clearly functions as an FEO, as observed in both fish and mammals, since a regular feeding schedule can rapidly entrain hepatic clock gene expression [14,19,20,21]. When food is provided during the rest period, there is a phase shift in the liver oscillator, to the extent that it becomes uncoupled with the hypothalamic one [16,22,23,24]. In goldfish, the hepatic clock is so sensitive to feeding that even a single meal can reset the hepatic clock, at least in the absence of the LD cycle [21]. However, despite many efforts taken, the mechanisms underlying feeding entrainment of the liver are still unclear [8,25,26,27].

Insulin is a well-known hypoglycemic and anabolic hormone secreted by the pancreatic β-cells in response to feeding, that binds to tyrosine-kinase insulin receptors in multiple target tissues [28]. In vertebrates including teleosts, the two main pathways of insulin signaling are the phosphatidylinositol 3-kinase (PI3K)/AKT (also known as PKB or protein kinase B) and the Raf/Ras/MEK/MAPK (mitogen-activated protein kinase, also known as ERK or extracellular signal regulated kinase) pathways, both of which have immediate effects over cell metabolism and gene expression [29]. The fact that the liver is highly sensitive to insulin signaling makes this hormone a good candidate as a mediator of feeding entrainment in this organ. This hypothesis gains support from research indicating that insulin resistance impairs the mammalian liver clock [30], and the deficiency of insulin or insulin receptors dampens feeding entrainment [31,32]. Accordingly, a handful of studies conducted in mammals have documented the effects of insulin on the liver oscillator, which immediately enhances the expression of *per* genes from the negative limb, and, thus, causes a phase-advance of the core clock rhythm [31,33,34,35,36]. To our knowledge, the effects of insulin over clock gene expression have not been reported yet in any fish species. 

Hence, the aim of this study was to analyze whether insulin may be involved in the entrainment of the liver clock by feeding in fish. This was achieved by examining the direct effects of insulin on clock gene expression in this organ, exploring its potential interaction with glucocorticoid-mediated entrainment, and the signaling pathways that may be involved.

## 2. Results

### 2.1. Insulin Treatment Enhances mRNA Abundance of per1a and per2 in the Liver of Goldfish

**Figure 1:**
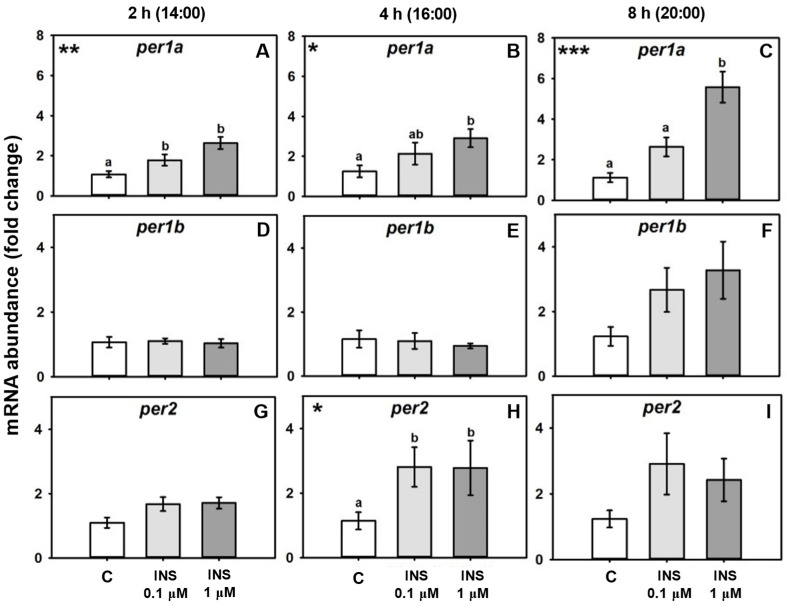
Dose–response effects of insulin treatment on per gene mRNA abundance. Relative mRNA abundance of the clock genes *per1a*, *per1b*, and *per2* in cultured goldfish livers treated with different concentrations of insulin for 2 h (**A**,**D**,**G**), 4 h (**B**,**E**,**H**), or 8 h (**C**,**F**,**I**). Data are shown as mean ± SEM; *n* = 8. Data were analyzed by one-way ANOVA (* *p* < 0.05; ** *p* < 0.005; *** *p* < 0.001). Significant differences among groups according to SNK test are represented by different letters.

Treatment of goldfish liver explants with insulin induced *per1* mRNA abundance in a dose- and time-dependent manner, almost reaching a 6-fold mRNA increase with the higher dose (1 µM) after 8 h (h) of treatment (Figure 1A–C). However, mRNA abundance of *per1b* was not significantly modified by insulin, and only tended to increase after 8 h of treatment (Figure 1D–F). The clock gene per2 was also upregulated by insulin, showing a significant increase at 4 h post-treatment (Figure 1G–I).

**Figure 2:**
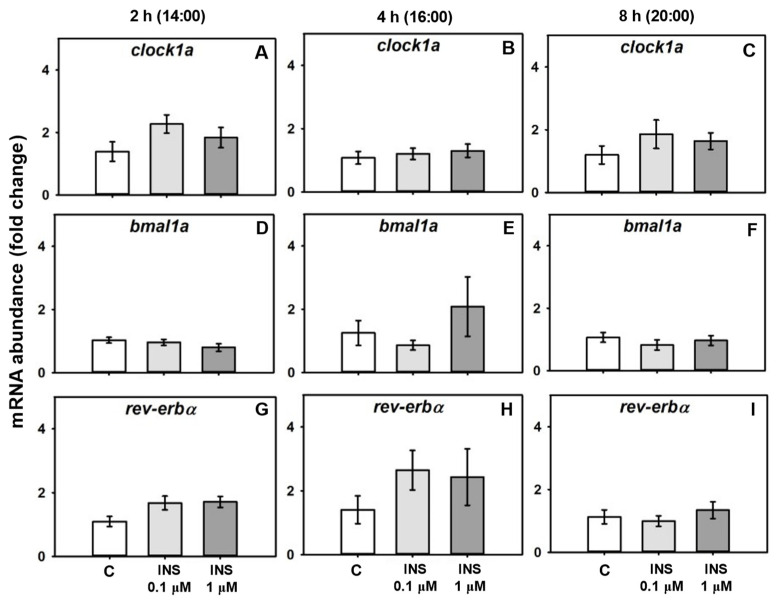
Dose–response effects of insulin treatment on clock gene mRNA abundance. Relative mRNA abundance of the clock genes *clock1a*, *bmal1a*, and *rev-erbα* in cultured goldfish liver treated with different concentrations of insulin for 2 h (**A**,**D**,**G**), 4 h (**B**,**E**,**H**), or 8 h (**C**,**F**,**I**). Data are shown as mean ± SEM; *n* = 8. Data were analyzed by one-way ANOVA.

The transcription of *rev-erbα*, *clock1a*, and *bmal1a* was not modified by insulin treatment (Figure 2).

### 2.2. Insulin Treatment Enhances mRNA Abundance of per Genes Independently of Protein Translation

**Figure 3:**
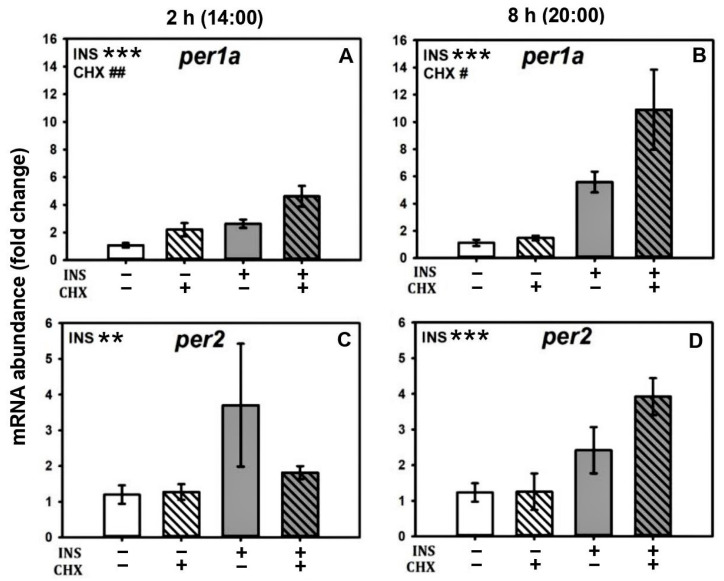
Effects of cycloheximide on insulin action over per mRNA abundance. Relative mRNA abundance of clock genes *per1a* and *per2* in cultured goldfish liver treated with 1 µM insulin or 10 µM cycloheximide alone or both drugs together for 2 h (**A**,**C**) or 8 h (**B**,**D**). Data are shown as mean ± SEM; *n* = 8. Data were analyzed by two-way ANOVA. INS = insulin. CHX = cycloheximide; ** *p* < 0.005 INS; *** *p* < 0.001 INS. # *p* < 0.05 CHX; ## *p* < 0.005 CHX.

Cycloheximide (a protein translation inhibitor) alone augmented mRNA abundance of per1a transcripts after 2 and 8 h of treatment (Figure 3A,B). Also, *per1a* and *per2* mRNA levels were increased by insulin treatment, and this induction was not affected by the treatment with cycloheximide, as no statistical interaction was found between the two factors (Figure 3A–D). Thus, the higher abundance of *per1a* and *per2* mRNA in the group treated with cycloheximide and insulin at 8 h post-treatment seem to result from the additive effects of both treatments in both cases (Figure 3B,D).

### 2.3. Insulin and Dexamethasone Have Independent Effects over per1a and per2 mRNA Abundance

**Figure 4:**
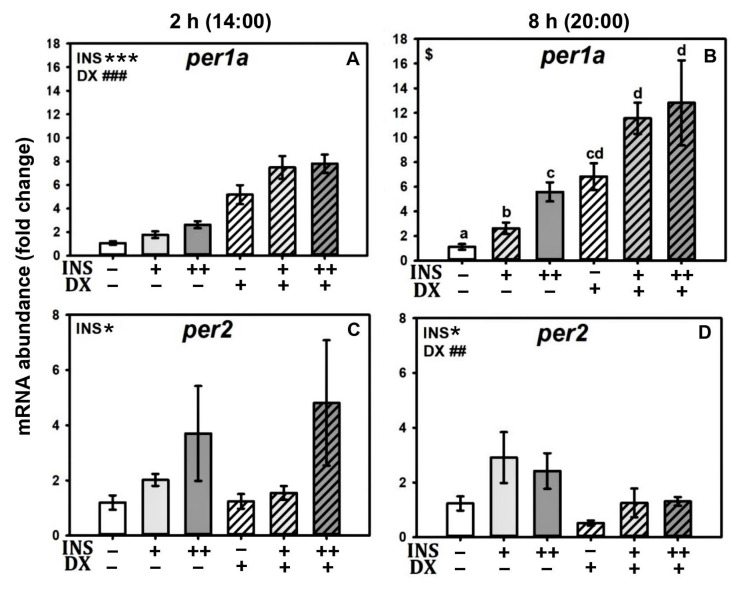
Effects of dexamethasone on insulin action over per mRNA abundance. Relative mRNA abundance of clock genes *per1a* and *per2* in cultured goldfish liver, treated with 0.1 µM insulin (+), 1 µM insulin (++), and 0.1 µM dexamethasone alone or both drugs together for 2 h (**A**,**C**) or 8 h (**B**,**D**). Data are shown as mean ± SEM; *n* = 8. Data were analyzed by two-way ANOVA. When an interaction between factors occurred, a one-way ANOVA followed by a SNK test was performed, and differences among groups were represented by different letters (**B**). INS = insulin. DX = dexamethasone. * *p* < 0.05 INS; *** *p* < 0.001 INS. ## *p* < 0.005 DX; ### *p* < 0.001 DX. $ interaction between drugs (*p* < 0.05).

Both insulin and dexamethasone alone caused an increase in *per1a* mRNA abundance after both 2 and 8 h (Figure 4A,B). The response to dexamethasone (6-fold increase) was similar at both treatment times, while the response to insulin increased with treatment duration (3-fold to 6-fold, Figure 4A,B). The two drugs exerted their effects independently after a 2 h treatment (Figure 4A). After 8 h, a statistical interaction occurred between DX and INS treatments, as the amount of per1a mRNA in the groups treated with both drugs was statistically lower than the sum of the separate effects (Figure 4B), which was probably the consequence of having reached a plateau in the transcription of the gene. 

As in the previous experiments, insulin treatment induced *per2* mRNA abundance after 2 and 8 h (Figure 4C,D). In contrast, dexamethasone treatment did not increase mRNA abundance of *per2*, and in fact caused a decrease in *per2* levels after 8 h. No interaction occurred between the effects of insulin and dexamethasone on *per2* mRNA abundance.

### 2.4. Insulin-Mediated Induction of per Genes Is Reversed by the Inactivation of the PI3K Pathway

**Figure 5:**
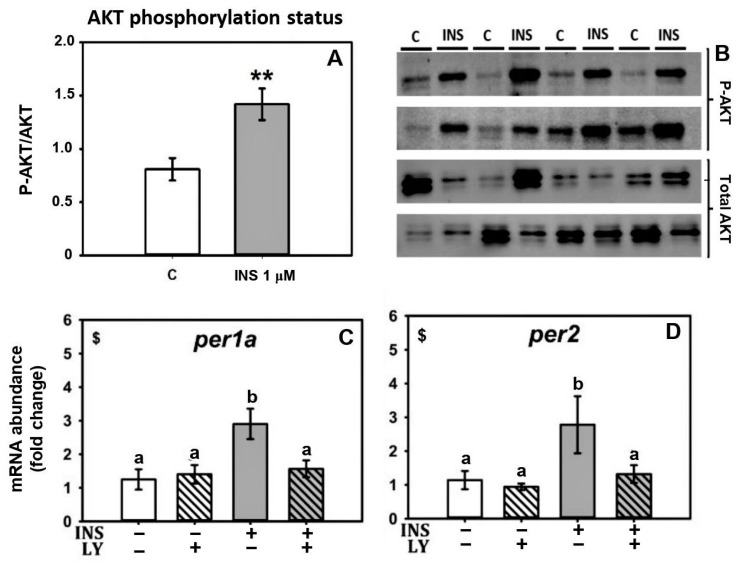
Involvement of PI3K/AKT pathway in insulin action on clock gene mRNA abundance. Western blot analysis of the phosphorylation status of AKT protein in cultured liver of goldfish after 4 h of treatment with control medium (**C**) or 1 µM insulin (INS). The graph (**A**) represents the p-AKT/AKT ratio from Western blots performed on 8 individual samples per treatment (mean ± SEM); and (**B**) shows representative blots. ** indicates significant differences (*p* < 0.005) between the groups according to Student’s *t*-test. (**C**,**D**) Effect of the PI3K/Akt pathway inhibitor LY294002 on insulin action over per1a and *per2* mRNA abundance (mean ± SEM; *n* = 8). Data were analyzed by two-way ANOVA. When an interaction between factors occurred, a one-way ANOVA followed by a SNK test was performed, and differences among groups were represented by different letters. INS = insulin. LY = LY294002. $ interaction between factors (*p* < 0.05).

The treatment with 1 µM insulin significantly increased AKT phosphorylation status, as shown by the enhanced *p*-AKT/AKT ratio, confirming the activation of the main insulin-signaling pathway (Figure 5A,B). Moreover, the insulin *per1a* and *per2* induction was abolished in the liver explants pre-treated with PI3K inhibitor LY294002 (Figure 5C,D). Although in the case of *per2* the interaction was slightly below significance, one-way ANOVA revealed that *per2* mRNA abundance in the double treatment was similar to the control group, i.e., insulin treatment had no effect in the presence of the inhibitor.

### 2.5. Insulin-Mediated Induction of per1a and per2 Is Independent of the MEK Pathway

**Figure 6:**
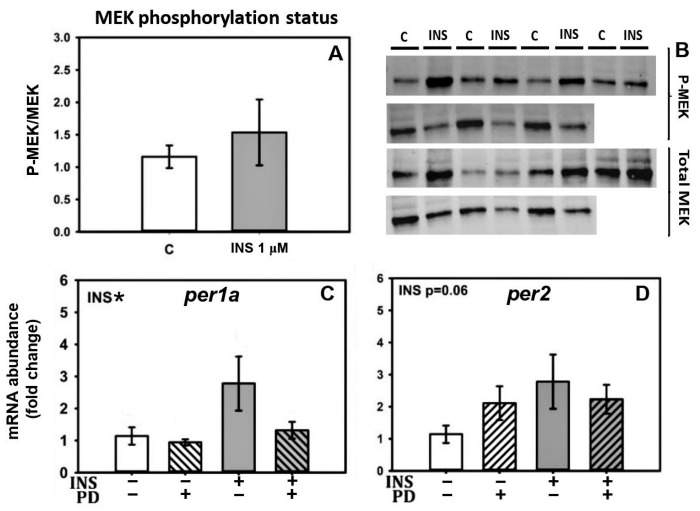
Involvement of the MEK pathway in insulin action on clock gene expression: (**A**,**B**) Western blot analysis of the phosphorylation status of MEK protein in cultured liver of goldfish after 4 h of treatment with control medium or 1 µM insulin (INS). The graph (**A**) represents the p-MEK/MEK ratio of Western blots performed on 7 individual samples per treatment (mean ± SEM); (**B**) shows representative blots. (**C**,**D**) Effect of the MEK pathway inhibitor PD98059 on insulin action over *per1a* and *per2* mRNA abundance (mean ± SEM; *n* = 8). Data were analyzed by two-way ANOVA (* *p* < 0.05 for insulin factor). INS = insulin. PD = PD95059.

Regarding the MEK pathway, there was no changes in MEK phosphorylation status after a 4 h treatment with 1 µM insulin based on the absence of changes in the p-MEK/MEK ratio (Figure 6A,B). In agreement, the inhibitor of the MEK pathway PD98059 did not interfere in the induction of *per1a* and *per2* by insulin treatment (Figure 6C,D).

## 3. Discussion

The results of this study provide insight into a potential mechanism for the feeding-induced resetting of the liver clock in teleost fish. Previous research has demonstrated the remarkable reliance of the endogenous hepatic oscillator on feeding time in goldfish [21,22] and rodents [20], but the precise link between food intake and clock gene rhythms in the liver remains uncertain. Insulin could be a potential mediator between both processes, considering that its levels increase after meals [28], and recent reports indicating the necessity of the insulin receptor for liver resetting in mice [32]. In this line, the present results provide evidence supporting the idea that insulin enhances mRNA abundance of *per1a* and *per2* genes in goldfish, suggesting that insulin plays a conserved role as an input for the liver clock in vertebrates. The induction of *per1a* in goldfish liver treated with insulin ex vivo is comparable with results obtained in mice, where insulin-mediated *per1* induction was observed both in vivo and in vitro [33,35,36]. This, along with the finding that the feeding time determines the phase of the *per1a* rhythm in the liver of teleosts [22,37], reinforces that postprandial insulin may be involved in this entrainment. Additionally, *per2* mRNA levels in goldfish liver increased significantly after 4 h of insulin treatment. In this line, a transient increase and phase advance of *per2* in response to insulin is well-documented in the mammalian liver clock [31,33,34,35], and a similar response is observed in other peripheral tissues [38,39,40]. Mice lacking the insulin receptor in hepatocytes also show altered *per2* entrainment by restricted feeding [32], further supporting that insulin-activated signaling pathways play an important role in this process. Notably, the induction of *per2* by insulin seems to be weaker in goldfish than in rodents. This could be due to a divergent role of *per2* between fish and mammals, as *per2* in teleosts is primarily considered a light-sensitive gene, mediating photic entrainment, as demonstrated in zebrafish tissues [41] and suggested for other teleost species [10]. However, the hypothesis that *per2* is light-induced does not exclude its regulation by feeding-related pathways, as the expression of this gene is also influenced by other feeding-related hormones linked to food intake, such as ghrelin [27]. Considering that the effect of insulin on *per* genes in mammals is not limited to induction, but also generates a phase advance that resets the hepatic clock [34], the presence of a similar mechanism seems likely in teleost fish, but further studies are required to assess this hypothesis.

In mammals, some reports suggest that insulin downregulates *rev-erbα* expression [31,36,40], whereas no effect was observed in goldfish. This could represent another potential difference in the circadian response to insulin between mammals and teleosts, which should be further investigated. The mRNA abundance of *clock1a* and *bmal1a* genes was not significantly affected by insulin treatment either. However, we cannot discard that insulin could act on other important clock genes in different models.

The upregulation of *per* genes by insulin is probably mediated by the insulin receptors in fish liver, as happens in mammals [32]. In support of this notion, we observed the activation of the PI3K/AKT pathway, a key transduction pathway triggered by the insulin receptor [29], in goldfish livers treated with insulin for 4 h. This was evidenced by the increased phosphorylation status of AKT [42]. Furthermore, pre-treating the goldfish liver with LY294002, a PI3K inhibitor, prevented *per1a* and *per2* induction by insulin. Similar findings have been reported in mammals, where LY294002 inhibits insulin-mediated PER2 increase in mouse fibroblasts and liver [33,34,38].

In contrast to the PI3K/AKT pathway, we did not observe significant changes in MEK phosphorylation in livers incubated with insulin, indicating that the MEK/ERK MAPK pathway was not activated by this hormone in the liver. These results are in line with others in rainbow trout muscle cells, where insulin failed to induce the MAPK pathway despite activating PI3K, suggesting that MAPK activation by insulin might be attenuated in the peripheral tissues of some adult fish species. Consistent with this, the MEK inhibitor PD98059 did not interfere with the induction of *per1a* and *per2* by insulin treatment in goldfish liver, suggesting that MEK activity is not required for this response. These results differ from a study in mice where PD98059 inhibited insulin-mediated *per1* induction [33]. However, in other studies, MEK pathway inhibitor U0126 had no significant effect on insulin entrainment in the liver or in fibroblasts [34,38], although it did in adipose tissue [34]. Thus, while in mammals the MEK pathway may be involved in part of the insulin actions over the molecular clock, in teleost fish the PI3K/AKT pathway appears to play a more significant role, at least in the liver.

The fact that the upregulation of *per1* and *per2* by insulin was not prevented by a pre-treatment with cycloheximide (present results) suggests that this response does not require intermediate protein translation. This supports the idea that the increase in the mRNA abundance of these transcripts is mediated by a mechanism directly activated by insulin signaling, which ends in transcription-inducing elements. To date it has not been possible to provide specific details regarding the precise mechanisms involved on such insulin actions. The first idea would be the existence of insulin-response elements found in the promoter regions of classical insulin-gene targets, like PEPCK and IRS-2, which bind forkhead proteins to regulate transcription in response to insulin signaling [43,44]. To our knowledge, no insulin-response element sequences have been identified in clock genes (including *per1*); thus, although we cannot rule out the presence of these insulin response elements, it seems unlikely. Another well-known response element related with light entrainment are the CRE elements and E-box sites that exist in all studied per promoters, and have a main role in endogenous clocks resetting [9,41]. However, these sites have not been linked to insulin effects on clock resetting to date. In fact, both sites have been excluded, at least for insulin actions on clock gene expression in adipose tissue of mammals [40]. To our knowledge the only transcription factors associated with insulin actions on the clock are NFY and SP1, which are activated by the PI3K/Akt pathway [40]. Considering that present data show that this pathway is involved in insulin action on clock genes, it is also likely to be the case in goldfish. Binding sites for NFY and SP1 transcriptions factor are present in per promoters in mammals, and their absence decreased insulin action on *per2* expression in adipose tissue of mammals [40]. In addition, other mechanisms of gene expression regulation have been revealed to also be important in mammals. Although the immediate increase in PER2 protein involves translation, a subsequent transcription-dependent mechanism appears to mediate it after the first 90 min [38]. Moreover, several potential signal transduction pathways have been proposed including the PI3K/AKT-mediated phosphorylation of BMAL1, resulting in its exclusion from the nucleus [36], as well as inhibition of *per1* and *per2* by regulatory microRNAs [38]. These putative mechanisms of insulin actions deserve to be explored in fish.

According to the present results, insulin seems to modulate hepatic clock genes, thereby reinforcing its role in the circadian system. There is substantial evidence supporting the relationship between insulin and the circadian system in vertebrates, including fish. For instance, insulin is a clock output synchronized to feeding schedule in teleosts [45]. Additionally, mistimed meals cause severe alterations in the circadian system and metabolism [22,46], consistent with the strong influence of insulin in the liver oscillator. In mammals, disruption of various clock genes causes hypoinsulinemia or hyperinsulinemia [47], and chronodisruption affects sensitivity to this hormone, supporting a strong link between clock gene expression and energy balance [47]. In this sense, insulin food entrainment may be required for coupling mealtimes with metabolic events maintaining optimal temporal homeostasis. However, under chronodisruptive conditions, this temporal homeostasis is lost, leading to a subsequently reduced welfare [17,22].

Nonetheless, in addition to insulin, other hormones and signaling molecules likely contribute to feeding entrainment in teleosts. Just like other crucial functions, such as feeding regulation, temporal homeostasis in the liver is probably regulated by multiple redundant signals. Specifically in goldfish, the orexigenic peptides orexin [26] and ghrelin [27,48], whose levels increase during fasting [49], can modulate the food anticipatory activity (FAA), which is the primary output of the food-entrainable oscillator (FEO) [26,27]. Unlike orexin and ghrelin, insulin levels increase directly in response to feeding [28,29]; thus, a stronger direct impact as a feeding input could be expected. Furthermore, several hormones apart from insulin directly affect clock gene expression in the fish liver, including ghrelin and also glucocorticoids, which will be further discussed [48,50]. Even more feeding-related signals have been implicated in liver clock entrainment in mammals, such as IGF-1 and oxyntomodulin (a hormone released by gastric filling), via induction of *per1* and *per2* [38,51]. Non-endocrine food entraining signals have also been described in mammals, such as redox state and sirtuins which seems to affect the circadian clock by modulating the activity of CLOCK and BMAL1 and/or deacetylating PER2 [52,53].

It is worth noting that many of the resetting signals of circadian clocks in vertebrates, including fish, act through modulation of the mRNA abundance of *per* genes. Now, insulin seems to be another of the numerous entraining cues that induce this family of genes in teleosts, along with light, glucocorticoids, orexin, and ghrelin [26,27,50]. In mammals, per genes have also been proposed as one of the food input sensors in the liver clock, as their expression responds acutely to nutrient intake and could initiate the adjustments in the oscillator machinery [54,55]. A similar situation seems to occur in fish, but it is not known how these different signals interact with each other in regulating *per* expression. Considering this, the present study sheds light on the combination of glucocorticoids as one of the stronger resetters of the clock of vertebrate peripheral tissues, including in fish [10,47,56,57], with the effects of insulin in the goldfish liver. The results provide further evidence of the role of glucocorticoids in regulating the hepatic circadian machinery of goldfish, and the fact that dexamethasone induced *per1a* ex vivo proves that the teleost liver oscillator is a direct target for glucocorticoid signaling [50]. This claim is supported by the abundance of glucocorticoid receptors on this organ and the known impact of glucocorticoids on the hepatic proteome [58]. However, even though glucocorticoid levels are modified by nutritional status [59] it does not seem likely that they serve as a primary feeding input of the liver clock, unlike insulin. This is evidenced by the persistence of hepatic circadian oscillations in mice lacking glucocorticoid receptors or adrenal glands [57,60]. Furthermore, when feeding time is inverted, the shifting of the liver core clock is not accompanied by a similar shift in the glucocorticoid rhythm in fish and mammals [22,35]. Along the same lines, other findings in mammals support the idea that hepatic synchronization by feeding and by endogenous glucocorticoids are independent, and that food intake is a stronger *Zeitgeber* for this organ [60].

Moreover, the present results suggest that the inductive effects of insulin over the *per* genes seem to be independent from the above-described glucocorticoid effects. This is indicated by the opposite effects of both hormones over *per2*, and the lack of statistically significant interaction between their actions. Thus, the effects of the combination of insulin and dexamethasone were equal to the effects of each of them separately, with the exception of the induction of *per1a* after 8 h of treatment, which could be attributed to having reached the limits of inductive capacity of this gene. These results allow us to suggest that glucocorticoids and insulin signal to the liver clock through different pathways, but further studies are required to assess this hypothesis. In this regard, the induction of *per* genes by glucocorticoids could be mediated by a putative glucocorticoid response element (GRE) sequence in the *per* gene promotor, as described in mice [61,62]. This mechanism is not expected to interfere with the PI3K/AKT pathway, which we proposed for insulin action on clock genes.

Interestingly, *per1a* rhythm in vivo shows a higher amplitude compared to in vitro conditions in the goldfish liver, even when this oscillator is reset with dexamethasone [63,64]. This observation, combined with the present results, supports the notion that a combination of multiple signals occurs in vivo, enhancing the robustness of oscillations [64]. Therefore, the present study reinforces the idea that the phase of the liver clock depends on the interplay among several input molecules.

Overall, the results show that insulin is a synchronizer for the liver clock, having direct effects over *per1* and *per2* clock genes in fish. This action does not require the translation of any intermediate proteins and might be mediated by the PI3K/AKT pathway. Therefore, the present work shows for the first time that insulin has the potential to regulate the positive limb of the circadian clock in the liver of teleosts, supporting its role as an important element of the probably multifactorial food entrainment mechanism of the fish hepatic oscillator.

## 4. Materials and Methods

### 4.1. Animals and Housing

Juvenile goldfish (*Carassius auratus*) of 15 ± 0.6 g body weight (bw), acquired from a local commercial supplier (ICA, Madrid, Spain), were kept in 60-liter tanks (*n* = 6–10 fish per tank) with environmental enrichment, and filtered and aerated fresh water (21 ± 1 °C) under a 12L:12D photoperiod (lights on at 8:00 h), for at least 15 days before the experiments. Food pellets (1% bw, Sera Pond Biogranulat, Heisenberg, Germany) were provided daily at 10:00 h (at ZT = 2, i.e., 2 h after lights on). All procedures complied with the Guidelines of the European Union Council (UE63/2010) and the Spanish Government (RD53/2013 and RD118/2021) for the use of animals in research and were approved by the Community of Madrid (PROEX 107/20).

### 4.2. Drugs

Insulin from bovine pancreas (Sigma-Aldrich, Madrid, Spain) was dissolved in pH 2–3 HCl-water, to a 1 mM concentration, on the same day of the experiment. Then, it was diluted in a control culture medium (Dulbecco’s Modified Eagle’s Medium, DMEM, Sigma-Aldrich) adapted to fish osmolarity as previously described [50], to reach 1 and 0.1 μM insulin concentrations (the final pH of these solutions was 7.4 as in controls). A stock solution of cycloheximide (Sigma-Aldrich), a protein translation inhibitor, was prepared in ethanol at 10 mM, and stored at 4 °C until the day of the experiment, in which it was diluted in DMEM to obtain the final concentration of 10 μM. A stock solution of 100 μM dexamethasone (Sigma-Aldrich) was first prepared in 3% ethanol distilled water and stored at 4 °C. The day it was used, dexamethasone stock solution was diluted sequentially in the culture medium until it reached 0.1 μM. LY294002 (PI3K inhibitor, TOCRIS, Bristol, UK) was dissolved into DMSO at 25 mM before the culture, and then diluted in culture medium to 50 µM, with DMSO reaching a final concentration of 0.2%. PD95059 (TOCRIS) was dissolved in 7.5 mM DMSO, and then diluted in 30 µM DMEM, with a DMSO final concentration of 0.4%.

### 4.3. Primary Hepatic Cultures

Liver cultures were performed as previously described [48,50]. In short, on the day of the experiments, non-fed goldfish were sacrificed by anesthetic overdose with MS-222 (tricaine methanesulfonate, 0.3 g/L, Sigma-Aldrich) at 10:00 h. Then, the liver was quickly sampled, homogenized using a scalpel, and distributed in different wells (≈20 mg liver/well) of sterile culture 24-well multi-dish plates. Each explant was pre-incubated in 1 mL DMEM, with penicillin–streptomycin (10 mL/L, Sigma-Aldrich,) and gentamycin (500 mg/L Sigma-Aldrich) for 2 h (21 °C, 3% CO_2_). After pre-incubation, the old medium was removed, fresh medium with the different treatments was added, and plates were introduced again into the incubator. In each experiment, the corresponding vehicle (i.e., DMSO or ethanol at final concentrations) was added to the control group. When the time of incubation was over, the whole content of the wells was recovered, the tissue was retrieved by soft centrifuging, and then frozen and stored at −80 °C until use.

### 4.4. Experimental Designs

#### 4.4.1. Insulin Effects on Hepatic Clock Gene Expression

The modulatory effect of insulin treatment on the mRNA abundance of different clock genes was studied. After pre-incubation in the control medium, liver aliquots from 8 goldfish were divided into 3 experimental groups (*n* = 8 explants/group): a control group, a group treated with 0.1 μM insulin (INS), and a group treated with 1 μM INS. Explants were retrieved after either 2, 4, or 8 h of treatment, and tissues were recovered, frozen, and stored for the analysis of mRNA abundance of clock genes (*per1a*, *per2*, *per1b*, *clock1a*, *rev-erbα*, *bmal1a*) by qPCR.

#### 4.4.2. Transcription Dependence of Insulin Action on Clock Genes

To know if the effects of insulin on the liver mRNA abundance of *per* genes were direct or mediated by intermediate transcription regulators, livers were extracted (*n* = 8) and divided into 4 experimental groups (*n* = 8 explants/group): vehicle (control), 1 µM insulin (INS), 10 µM cycloheximide (CHX), and 10 µM cycloheximide plus 1 µM insulin (INS/CHX). In the group combining CHX and INS, CHX was added 30 min before insulin to block transcription. The treatments were maintained for either 2 or 8 h of incubation. Then, tissues were recovered, frozen, and stored until qPCR analysis (*per1a* and *per2*).

#### 4.4.3. Possible Interaction between Dexamethasone and Insulin Treatments on *per1* and *per2* Induction

To evaluate a possible interaction between insulin and dexamethasone clock gene induction in the liver oscillator, 8 goldfish livers were cultured as indicated in Section 4.3. After the preincubation, 6 different treatments were employed (*n* = 8 explants/group): vehicle (control), 0.1 μM insulin (INS+), 1 μM insulin (INS++), 0.1 μM dexamethasone (DX), 0.1 μM insulin and 0.1 μM dexamethasone (INS+/DX), and 1 μM insulin and 0.1 μM dexamethasone (INS++/DX). Both drugs (insulin and dexamethasone) were added to the explants at the same time. After either 2 or 8 h of treatment, tissues were recovered, frozen, and stored for qPCR gene expression analysis (*per1a* and *per2*).

#### 4.4.4. Second Messenger Pathways Activated by Insulin in Goldfish Cultured Liver

To determine if insulin activates its canonical signaling pathways in goldfish liver, the activation of PI3K/AKT and MEK/ERK pathways was analyzed by Western blotting through assessment of the phosphorylation status of AKT and MEK. Liver cultures were performed as described in Section 2.3, but with a higher amount of tissue (≈40 mg). Liver explants were extracted from 8 goldfish and divided into the control group and 1 µM insulin group (INS), *n* = 8 explants/group. After 4 h, explants were cryogenized and stored for Western blot analysis of the p-AKT/AKT and p-MEK/MEK ratios.

#### 4.4.5. Involvement of PI3K and MEK/ERK Pathways on Insulin-Mediated *per1* and *per2* Induction

To study whether insulin’s action on *per1* and *per2* was mediated by the activation of PI3K/AKT or MEK/ERK pathways, inhibitors of both routes were employed. Eight livers were used to perform the following experimental groups (*n* = 8 explants/group): vehicle, 1 μM insulin (INS), 50 µM LY294002 (LY), 50 µM LY294002 plus 1 μM insulin (INS/LY), 30 µM PD95809 (PD), and 30 µM PD95809 plus 1 μM insulin (INS/PD). After 4 h of treatment, the tissue was recovered, frozen, and stored for qPCR gene expression analysis (*per1a* and *per2*).

### 4.5. Western Blotting

Western blotting was used to assess the phosphorylation status of AKT and MEK through quantification of p-AKT/AKT and p-MEK/MEK ratios, as detailed in previous studies [65]. In short, frozen samples (around 20 mg) were homogenized in 1 mL of buffer containing 150 mM NaCl, 10 mM tris-HCl, 1 mM EGTA, 1 mM EDTA (pH 7.4), 100 mM sodium fluoride, 4 mM sodium pyrophosphate, 2 mM sodium orthovanadate, 1% Triton X-100, 0.5% NP40-IGEPAL, and 1.02 mg/mL protease inhibitor cocktail (Sigma-Aldrich) via sonication, keeping the tubes on ice to prevent denaturation. Homogenates were centrifuged twice (1000× *g*, 15 min; 1000× *g*, 30 min), retrieving the supernatant each time. The concentration of protein in each sample was determined by the Bradford method, using a standard curve with bovine serum albumin. Then, protein lysates were denaturized at 95 °C for 5 min with β-mercaptoethanol and loading dye, and 20 µg of each sample was loaded in a 10% acrylamide gel and Western blotted. The primary antibodies employed were 1:1000 Anti-phospho-AKT (Ser 473) (cs #4060, Cell Signaling, Danvers, MA, USA), 1:1000 anti-AKT (cs #9272, Cell Signaling), 1:500 anti-phospho-MEK1/2 (Ser217/221) (cs #9154, Cell Signaling), and 1:500 anti-MEK1/2 (cs#9122, Cell Signaling). The secondary antibody used was anti-rabbit (1:10,000, ab205718, Abcam, Cambridge, UK). Bands were clean and matched the expected size of the protein detected. Band density was quantified by Image Lab software 6.0. (Bio-Rad Laboratories, Hercules, CA, USA) in a Chemidoc Touch Imaging System (Bio-Rad Laboratories).

### 4.6. Assessment of mRNA Abundance by RT-qPCR

Total RNA from the liver explants was isolated by the TRIzol/chloroform method, using TRI Reagent (Sigma-Aldrich), and treated with RQ1 RNAse-free DNAse (Promega, Madison, WI, USA). Then, 0.3 µg of total RNA was reverse-transcribed into cDNA in a 25 µL reaction volume, using random primers (Invitrogen, Waltham, MA, USA), RNAse inhibitors (RNAsin, Promega), and SuperScript II Reverse Transcriptase (Invitrogen). RT-qPCR was carried out in each sample in duplicate using iTaq Universal SYBR Green Supermix (Bio-Rad Laboratories) into a 96-well plastic plates loaded with a CFX96TM Real-Time System (Bio-Rad Laboratories), with 1 µL of cDNA and a final concentration of 0.5 µM of each forward and reverse primers, to a final volume of 10 µL. The GeneBank reference number and primer (Sigma-Aldrich) sequences are shown in Table 1. The cycling protocol included a first step of denaturation (95 °C, 30 s), and 40 cycles of amplification (95 °C for 5 s, and 60 °C for 30 s). At the end of every amplification protocol, a temperature gradient (from 70 to 90 °C, at a rate of 0.5 °C/5 s) was applied in order to obtain melting curves that helped confirm the specificity of the reaction. Non-retrotranscribed RNA and water were run as controls. cDNA standard curves were analyzed to ensure optimal amplification conditions (efficiency = 90–105%). Relative mRNA abundance was determined by the 2^−ΔΔCt^ method [66]. Data obtained were normalized to the control group.

### 4.7. Statistics

For all statistical analyses and representations, Sigmaplot 12.0 software was used. First, the normality and homoscedasticity of the data sets were tested using the Shapiro–Wilk test and Levene tests, respectively. When necessary, the data were transformed to logarithmic or square root scale in order to meet these requirements. For experiments with only one factor (e.g., different insulin doses), differences among groups were determined by one-way ANOVA followed by a Student–Newman–Keuls (SNK) post hoc test. When two treatments were combined, a two-way ANOVA was used to determine the effects of the two factors separately or a possible interaction between them. If a significant interaction was found, the groups were compared by one-way ANOVA followed by a Student–Newman–Keuls (SNK) post hoc test.

## Figures and Tables

**Table 1:** Primers used for RT-qPCR analysis.

Gene Transcript	Access Number (GenBank)	Sequence (5′→3′)		Product (bp)
***β-actin***	AB039726.2	Forward	CAGGGAGTGATGGTTGGCA	168
		Reverse	AACACGCAGCTCGTTGTAGA	
***bmal1a***	KF840401.1	Forward	AGATTCTGTTCGTCTCGGAG	161
		Reverse	ATCGATGAGTCGTTCCCGTG	
***clock1a***	KJ574204.1	Forward	CGATGGCAGCATCTCTTGTGT	187
		Reverse	TCCTGGATCTGCCGCAGTTCAT	
***per1a***	EF690698.1	Forward	CAGTGGCTCGAATGAGCACCA	155
		Reverse	TGAAGACCTGCTGTCCGTTGG	
***per1b***	KP663726.1	Forward	CTCGCAGCTCCACAAACCTA	235
		Reverse	TGATCGTGCAGAAGGAGCCG	
***per2***	EF690697.1	Forward	TTTGTCAATCCCTGGAGCCGC	105
		Reverse	AAGGATTTGCCCTCAGCCACG	
***rev-erbα***	KU242427	Forward	CGTTCATCTCAGGCACCACT	166
		Reverse	AACTGACCTGCAGACACCAG	

## Data Availability

Data are contained within the article.

## References

[1] Roenneberg T., Merrow M. (2003). The Network of Time: Understanding the Molecular Circadian System. Curr. Biol..

[2] Patke A., Young M.W., Axelrod S. (2020). Molecular Mechanisms and Physiological Importance of Circadian Rhythms. Nat. Rev. Mol. Cell Biol.

[3] Albrecht U. (2012). Timing to Perfection: The Biology of Central and Peripheral Circadian Clocks. Neuron.

[4] Vatine G., Vallone D., Gothilf Y., Foulkes N.S. (2011). It’s Time to Swim! Zebrafish and the Circadian Clock. FEBS Lett..

[5] Schibler U., Gotic I., Saini C., Gos P., Curie T., Emmenegger Y., Sinturel F., Gosselin P., Gerber A., Fleury-Olela F. (2015). Clock-Talk: Interactions between Central and Peripheral Circadian Oscillators in Mammals. Cold Spring Harbor Symposia on Quantitative Biology.

[6] Zhang R., Lahens N.F., Ballance H.I., Hughes M.E., Hogenesch J.B. (2014). A Circadian Gene Expression Atlas in Mammals: Implications for Biology and Medicine. Proc. Natl. Acad. Sci. USA.

[7] Cahill G.M. (2002). Clock Mechanisms in Zebrafish. Cell Tissue Res..

[8] Sánchez-Bretaño A., Alonso-Gómez Á.L., Delgado M.J., Isorna E. (2015). The Liver of Goldfish as a Component of the Circadian System: Integrating a Network of Signals. Gen. Comp. Endocrinol..

[9] Frøland Steindal I.A., Whitmore D. (2019). Circadian Clocks in Fish-What Have We Learned so Far?. Biology.

[10] Isorna E., de Pedro N., Valenciano A.I., Alonso-Gómez Á.L., Delgado M.J. (2017). Interplay between the Endocrine and Circadian Systems in Fishes. J. Endocrinol..

[11] West A.C., Iversen M., Jørgensen E.H., Sandve S.R., Hazlerigg D.G., Wood S.H. (2020). Diversified Regulation of Circadian Clock Gene Expression Following Whole Genome Duplication. PLoS Genet..

[12] Golombek D.A., Rosenstein R.E. (2010). Physiology of Circadian Entrainment. Physiol. Rev..

[13] Ashton A., Foster R.G., Jagannath A. (2022). Photic Entrainment of the Circadian System. Int. J. Mol. Sci..

[14] López-Olmeda J.F. (2017). Nonphotic Entrainment in Fish. Comp. Biochem. Physiol. A Mol. Integr. Physiol..

[15] Mendoza J. (2007). Circadian Clocks: Setting Time by Food. J. Neuroendocrinol..

[16] López-Olmeda J.F., Tartaglione E.V., De La Iglesia H.O., Sánchez-Vázquez F.J. (2010). Feeding Entrainment of Food-Anticipatory Activity and Per1 Expression in the Brain and Liver of Zebrafish under Different Lighting and Feeding Conditions. Chronobiol. Int..

[17] Saiz N., Gómez-Boronat M., De Pedro N., Delgado M.J., Isorna E. (2021). The Lack of Light-Dark and Feeding-Fasting Cycles Alters Temporal Events in the Goldfish (*Carassius auratus*) Stress Axis. Animals.

[18] Ferrell J.M., Chiang J.Y.L. (2015). Circadian Rhythms in Liver Metabolism and Disease. Acta Pharm. Sin. B.

[19] Pickel L., Sung H.K. (2020). Feeding Rhythms and the Circadian Regulation of Metabolism. Front. Nutr..

[20] Damiola F., Le Minli N., Preitner N., Kornmann B., Fleury-Olela F., Schibler U. (2000). Restricted Feeding Uncouples Circadian Oscillators in Peripheral Tissues from the Central Pacemaker in the Suprachiasmatic Nucleus. Genes Dev..

[21] Feliciano A., Vivas Y., De Pedro N., Delgado M.J., Velarde E., Isorna E. (2011). Feeding Time Synchronizes Clock Gene Rhythmic Expression in Brain and Liver of Goldfish (*Carassius auratus*). J. Biol. Rhythms..

[22] Gómez-Boronat M., Sáiz N., Delgado M.J., de Pedro N., Isorna E. (2018). Time-Lag in Feeding Schedule Acts as a Stressor That Alters Circadian Oscillators in Goldfish. Front. Physiol..

[23] Vera L.M., Negrini P., Zagatti C., Frigato E., Sánchez-Vázquez F.J., Bertolucci C. (2013). Light and Feeding Entrainment of the Molecular Circadian Clock in a Marine Teleost (*Sparus aurata*). Chronobiol. Int..

[24] Costa L.S., Serrano I., Sánchez-Vázquez F.J., López-Olmeda J.F. (2016). Circadian Rhythms of Clock Gene Expression in Nile Tilapia (*Oreochromis niloticus*) Central and Peripheral Tissues: Influence of Different Lighting and Feeding Conditions. J. Comp. Physiol. B.

[25] Bass J., Takahashi J.S. (2010). Circadian Integration of Metabolism and Energetics. Science.

[26] Nisembaum L.G., De Pedro N., Delgado M.J., Sánchez-Bretaño A., Isorna E. (2014). Orexin as an Input of Circadian System in Goldfish: Effects on Clock Gene Expression and Locomotor Activity Rhythms. Peptides.

[27] Nisembaum L.G., de Pedro N., Delgado M.J., Isorna E. (2014). Crosstalking between the “Gut-Brain” Hormone Ghrelin and the Circadian System in the Goldfish. Effects on Clock Gene Expression and Food Anticipatory Activity. Gen. Comp. Endocrinol..

[28] De Meyts P. (2000). The Insulin Receptor and Its Signal Transduction Network.

[29] Caruso M.A., Sheridan M.A. (2011). New Insights into the Signaling System and Function of Insulin in Fish. Gen. Comp. Endocrinol..

[30] Honma K., Hikosaka M., Mochizuki K., Goda T. (2016). Loss of Circadian Rhythm of Circulating Insulin Concentration Induced by High-Fat Diet Intake Is Associated with Disrupted Rhythmic Expression of Circadian Clock Genes in the Liver. Metabolism.

[31] Tahara Y., Otsuka M., Fuse Y., Hirao A., Shibata S. (2011). Refeeding after Fasting Elicits Insulin-Dependent Regulation of Per2 and Rev-Erbα with Shifts in the Liver Clock. J. Biol. Rhythms.

[32] Fougeray T., Polizzi A., Régnier M., Fougerat A., Ellero-Simatos S., Lippi Y., Smati S., Lasserre F., Tramunt B., Huillet M. (2022). The Hepatocyte Insulin Receptor Is Required to Program the Liver Clock and Rhythmic Gene Expression. Cell Rep..

[33] Yamajuku D., Inagaki T., Haruma T., Okubo S., Kataoka Y., Kobayashi S., Ikegami K., Laurent T., Kojima T., Noutomi K. (2012). Real-Time Monitoring in Three-Dimensional Hepatocytes Reveals That Insulin Acts as a Synchronizer for Liver Clock. Sci. Rep..

[34] Sato M., Murakami M., Node K., Matsumura R., Akashi M. (2014). The Role of the Endocrine System in Feeding-Induced Tissue-Specific Circadian Entrainment. Cell Rep..

[35] Oishi K., Yasumoto Y., Higo-Yamamoto S., Yamamoto S., Ohkura N. (2017). Feeding Cycle-Dependent Circulating Insulin Fluctuation Is Not a Dominant Zeitgeber for Mouse Peripheral Clocks except in the Liver: Differences between Endogenous and Exogenous Insulin Effects. Biochem. Biophys. Res. Commun..

[36] Dang F., Sun X., Ma X., Wu R., Zhang D., Chen Y., Xu Q., Wu Y., Liu Y. (2016). Insulin Post-Transcriptionally Modulates Bmal1 Protein to Affect the Hepatic Circadian Clock. Nat. Commun..

[37] Del Pozo A., Montoya A., Vera L.M., Sánchez-Vázquez F.J. (2012). Daily Rhythms of Clock Gene Expression, Glycaemia and Digestive Physiology in Diurnal/Nocturnal European Seabass. Physiol. Behav..

[38] Crosby P., Hamnett R., Putker M., Hoyle N.P., Reed M., Karam C.J., Maywood E.S., Stangherlin A., Chesham J.E., Hayter E.A. (2019). Insulin/IGF-1 Drives PERIOD Synthesis to Entrain Circadian Rhythms with Feeding Time. Cell.

[39] Kajimoto J., Matsumura R., Node K., Akashi M. (2018). Potential Role of the Pancreatic Hormone Insulin in Resetting Human Peripheral Clocks. Genes Cells.

[40] Tuvia N., Pivovarova-Ramich O., Murahovschi V., Lück S., Grudziecki A., Ost A.C., Kruse M., Nikiforova V.J., Osterhoff M., Gottmann P. (2021). Insulin Directly Regulates the Circadian Clock in Adipose Tissue. Diabetes.

[41] Vatine G., Vallone D., Appelbaum L., Mracek P., Ben-Moshe Z., Lahiri K., Gothilf Y., Foulkes N.S. (2009). Light Directs Zebrafish Period2 Expression via Conserved D and E Boxes. PLoS Biol..

[42] Siddle K. (2011). Signalling by Insulin and IGF Receptors: Supporting Acts and New Players. J. Mol. Endocrinol..

[43] Durham S.K., Suwanichkul A., Scheimann A.O., Yee D., Jackson J.G., Barr F.G., Powell D.R. (1999). FKHR Binds the Insulin Response Element in the Insulin-Like Growth Factor Binding Protein-1 Promoter. Endocrinology.

[44] Zhang J., Ou J., Bashmakov Y., Horton J.D., Brown M.S., Goldstein J.L. (2001). Insulin Inhibits Transcription of IRS-2 Gene in Rat Liver through an Insulin Response Element (IRE) That Resembles IREs of Other Insulin-Repressed Genes. Proc. Natl. Acad. Sci. USA.

[45] Gutierrez J., Carrillo M., Zanuy S., Planas J. (1984). Daily Rhythms of Insulin and Glucose Levels in the Plasma of Sea Bass *Dicentrarchus abrax* after Experimental Feeding. Gen. Comp. Endocrinol..

[46] Boege H.L., Bhatti M.Z., St-Onge M.P. (2021). Circadian Rhythms and Meal Timing: Impact on Energy Balance and Body Weight. Curr. Opin. Biotechnol..

[47] Tsang A.H., Barclay J.L., Oster H. (2013). Interactions between Endocrine and Circadian Systems. J. Mol. Endocrinol..

[48] Sánchez-Bretaño A., Blanco A.M., Alonso-Gómez Á.L., Delgado M.J., Kah O., Isorna E. (2017). Ghrelin Induces Clock Gene Expression in the Liver of Goldfish in Vitro via Protein Kinase C and Protein Kinase A Pathways. J. Exp. Biol..

[49] Soengas J.L., Cerdá-Reverter J.M., Delgado M.J. (2018). Central Regulation of Food Intake in Fish: An Evolutionary Perspective. J. Mol. Endocrinol..

[50] Sánchez-Bretaño A., Callejo M., Montero M., Alonso-Gómez Á.L., Delgado M.J., Isorna E. (2016). Performing a Hepatic Timing Signal: Glucocorticoids Induce *Gper1a* and *Gper1b* Expression and Repress *Gclock1a* and *Gbmal1a* in the Liver of Goldfish. J. Comp. Physiol. B.

[51] Landgraf D., Tsang A.H., Leliavski A., Koch C.E., Barclay J.L., Drucker D.J., Oster H. (2015). Oxyntomodulin Regulates Resetting of the Liver Circadian Clock by Food. Elife.

[52] Rutter J., Reick M., Wu L.C., McKnight S.L. (2001). Regulation of Clock and NPAS2 DNA Binding by the Redox State of NAD Cofactors. Science.

[53] Asher G., Gatfield D., Stratmann M., Reinke H., Dibner C., Kreppel F., Mostoslavsky R., Alt F.W., Schibler U. (2008). SIRT1 Regulates Circadian Clock Gene Expression through PER2 Deacetylation. Cell.

[54] Wu T., Fu O., Yao L., Sun L., Zhuge F., Fu Z. (2012). Differential Responses of Peripheral Circadian Clocks to a Short-Term Feeding Stimulus. Mol. Biol. Rep..

[55] Oike H., Oishi K., Kobori M. (2014). Nutrients, Clock Genes, and Chrononutrition. Curr. Nutr. Rep..

[56] Reddy A.B., Maywood E.S., Karp N.A., King V.M., Inoue Y., Gonzalez F.J., Lilley K.S., Kyriacou C.P., Hastings M.H. (2007). Glucocorticoid Signaling Synchronizes the Liver Circadian Transcriptome. Hepatology.

[57] Balsalobre A., Brown S.A., Marcacci L., Tronche F., Kellendonk C., Reichardt H.M., Schutz G., Schibler U. (2000). Resetting of Circadian Time in Peripheral Tissues by Glucocorticoid Signaling. Science.

[58] Vijayan M.M., Raptis S., Sathiyaa R. (2003). Cortisol Treatment Affects Glucocorticoid Receptor and Glucocorticoid-Responsive Genes in the Liver of Rainbow Trout. Gen. Comp. Endocrinol..

[59] Woodward C.J.H., Hervey G.R., Oakey R.E., Whitaker E.M. (1991). The Effects of Fasting on Plasma Corticosterone Kinetics in Rats. Br. J. Nutr..

[60] Sujino M., Furukawa K., Koinuma S., Fujioka A., Nagano M., Iigo M., Shigeyoshi Y. (2012). Differential Entrainment of Peripheral Clocks in the Rat by Glucocorticoid and Feeding. Endocrinology.

[61] Yamamoto T., Nakahata Y., Tanaka M., Yoshida M., Soma H., Shinohara K., Yasuda A., Mamine T., Takumi T. (2005). Acute Physical Stress Elevates Mouse Period1 MRNA Expression in Mouse Peripheral Tissues via a Glucocorticoid-Responsive Element. J. Biol. Chem..

[62] So A.Y.-L., Bernal T.U., Pillsbury M.L., Yamamoto K.R., Feldman B.J. (2009). Glucocorticoid Regulation of the Circadian Clock Modulates Glucose Homeostasis. Proc. Natl. Acad. Sci. USA.

[63] Isorna E., Sánchez-Bretaño A., Nisembaum L.G., Alonso-Gómez Á.L., de Pedro N., Delgado M.J. (2018). Hormonal Inputs for Fish Circadian System: Goldfish as a Model. Abstracts of the XXXIX Congress of the Spanish Society of Physiological Sciences.

[64] Sánchez-Bretaño A. (2017). Interacción Entre El Sistema Endocrino y Los Osciladores Circadianos En El Carpín (*Carassius auratus*): Regulación de Genes Reloj En Relojes Centrales y Periféricos Por Péptidos de Origen Gastrointestinal y Por Glucocorticoides. Ph.D. Thesis.

[65] Otero-Rodino C., Velasco C., Álvarez-Otero R., López-Patino M.A., Miguez J.M., Soengas J.L. (2017). Changes in the Levels and Phosphorylation Status of Akt, AMPK, CREB and FoxO1 in Hypothalamus of Rainbow Trout under Conditions of Enhanced Glucosensing Activity. J. Exp. Biol..

[66] Livak K.J., Schmittgen T.D. (2001). Analysis of Relative Gene Expression Data Using Real-Time Quantitative PCR and the 2^−ΔΔCT^ Method. Methods.

